# Non-invasive Maternal Hemodynamic Assessment to Classify High-Risk Pregnancies Complicated by Fetal Growth Restriction

**DOI:** 10.3389/fcdhc.2022.851971

**Published:** 2022-05-04

**Authors:** Sara Ornaghi, Andrea Caricati, Daniela Denis Di Martino, Martina Mossa, Sara Di Nicola, Francesca Invernizzi, Sara Zullino, Sara Clemenza, Valentina Barbati, Gabriele Tinè, Federico Mecacci, Enrico Ferrazzi, Patrizia Vergani

**Affiliations:** ^1^ Department of Obstetrics and Gynecology, Unit of Obstetrics, Monza e Brianza per il Bambino e la sua Mamma Foundation Onlus at San Gerardo Hospital, Monza, Italy; ^2^ University of Milan-Bicocca School of Medicine and Surgery, Monza, Italy; ^3^ Department of Obstetrics and Gynecology, Unit of Obstetrics, Department of Woman, Child, and Newborn, Fondazione IRCCS Ca’ Granda – Ospedale Maggiore Policlinico, Milan, Italy; ^4^ Department of Obstetrics and Gynecology, Biomedical, Experimental and Clinical Sciences, University Hospital Careggi, Florence, Italy; ^5^ Department of Economics and Quantitative Methods, University of Milan-Bicocca, Monza, Italy; ^6^ Department of Clinical and Community Sciences, University of Milan, Milan, Italy

**Keywords:** Fetal growth restriction (FGR), hemodynamic, USCOM-1A, pregnancy, hypertension, gestational age

## Abstract

**Objectives:**

To verify whether the use of the temporal criterion of 32 weeks’ gestation is effective in identifying maternal hemodynamic differences between early- and late-onset fetal growth restriction (FGR), and to test the statistical performance of a classificatory algorithm for FGR.

**Materials and methods:**

A prospective multicenter study conducted at three centers over 17 months. Singleton pregnant women with a diagnosis of FGR based on the international Delphi survey consensus at ≥ 20 weeks of gestation were included. FGR was classified as early-onset if diagnosed <32 weeks’ gestation and as late-onset if ≥32 weeks. Hemodynamic assessment was performed by USCOM-1A at the time of FGR diagnosis. Comparisons between early- and late-onset FGR among the entire study cohort, FGR associated with hypertensive disorders of pregnancy (HDP-FGR), and isolated FGR (i-FGR) were performed. In addition, HDP-FGR cases were compared to i-FGR, regardless of the temporal cut-off of 32 weeks’ gestation. Finally, a classificatory analysis based on the Random Forest model was performed to identify significant variables with the ability to differentiate FGR phenotypes.

**Results:**

During the study period, 146 pregnant women fulfilled the inclusion criteria. In 44 cases, FGR was not confirmed at birth, thus limiting the final study population to 102 patients. In 49 (48.1%) women, FGR was associated to HDP. Fifty-nine (57.8%) cases were classified as early-onset. Comparison of the maternal hemodynamics between early- and late-onset FGR did not show any difference. Similarly, non-significant findings were observed in sensitivity analyses performed for HDP-FGR and for i-FGR. In turn, comparison between pregnant women with FGR and hypertension and women with i-FGR, independently of the gestational age at FGR diagnosis, revealed substantial differences, with the former showing higher vascular peripheral resistances and lower cardiac output, among other significant parameters. The classificatory analysis identified both phenotypic and hemodynamic variables as relevant in distinguishing HDP-FGR from i-FGR (p=0.009).

**Conclusions:**

Our data show that HDP, rather than gestational age at FGR diagnosis, allows to appreciate specific maternal hemodynamic patterns and to accurately distinguish two different FGR phenotypes. In addition, maternal hemodynamics, alongside phenotypic characteristics, play a central role in classifying these high-risk pregnancies.

## Introduction

Fetal growth restriction (FGR) is defined as the failure of the fetus to reach its genetically determined growth potential, and it occurs in about 10% of pregnancies (1).

The identification of growth restricted fetuses during gestation is crucial to ensure optimal monitoring and timing of birth, thus possibly reducing the high risk of stillbirth and perinatal morbidity associated with this condition (2, 3).

Currently, the most recognized criteria to define FGR are those derived from an international Delphi survey consensus (4). These criteria include biometric parameters and Doppler indices of feto-placental function, which allow to classify FGR into two phenotypes, early- and late-onset, according to the timing of diagnosis with a 32-gestational weeks cut-off. These phenotypes differ significantly in many aspects, such as prevalence, perinatal outcomes, and concomitant maternal diseases. Particularly, early-onset FGR is frequently associated with hypertensive disorders of pregnancy (HDP), including gestational hypertension and preeclampsia, whereas late-onset FGR is less related to such obstetric complications.

Interestingly, FGR has been considered for a long time to be solely the consequence of placental dysfunction or insufficiency (5). However, in recent years, a potential relationship between FGR and inadequate maternal systemic hemodynamic adaptation to pregnancy has been suggested (6–15). Currently, an integrated maternal hemodynamic-placental model is considered as the most appropriate and exhaustive for explaining the pathogenetic mechanisms underlying FGR (3, 16). Of note, the recently published practice guidelines on FGR by the ISUOG have highlighted the key role of the maternal hemodynamic status in differentiating early- and late-onset FGR, identifying more marked cardiovascular abnormalities, such as low cardiac output and high peripheral vascular resistance, among early-onset cases (3).

Previous research works on maternal hemodynamics among women with HDP have shown that the association with FGR, rather than the gestational age at disease onset with the traditional 34-gestational weeks cut-off, more clearly defines different HDP phenotypes (11, 17–19).

In contrast, similar studies among pregnancies complicated by FGR using the 32-gestational weeks cut-off as proposed by the international Delphi survey consensus and the ISUOG guidelines (3, 4) are lacking. Thus, the aim of our work was to investigate whether such temporal criterion would be valid in accurately differentiating the two proposed FGR phenotypes, early- and late-onset, specifically focusing on the accompanying maternal hemodynamic status.

## Methods

This was an observational, prospective multicenter study conducted at the Obstetric Unit of three Italian university, maternal-fetal referral centers in Lombardy and Tuscany (Milan, Monza and Florence) over a continuous period of time from November 2019 to April 2021.

Written informed consent was acquired from all study participants and research ethics committee approval (University of Milan-Bicocca, IRB n. 2988, approved on December 13, 2018) was obtained prior to the start of study investigations.

The inclusion criteria were singleton pregnancy with a viable fetus at ≥ 20 weeks of gestation and a diagnosis of FGR based on the international Delphi survey consensus (4). FGR cases could be isolated or in association with HPD (gestational hypertension or preeclampsia), as defined by the ISSHP classification (20). The exclusion criteria were multiple gestation, fetal aneuploidy, genetic syndromes or major structural fetal anomalies, preterm rupture of membranes, intrauterine infection, undetermined gestational age, maternal infection, maternal chronic hypertension or heart disease, maternal kidney or autoimmune disease, and use of antihypertensive drugs before enrollment.

After delivery, neonates of recruited women were assessed for biometric parameters, and only those women with growth-restricted neonates, as defined by a Delphi consensus procedure *in utero* (21, 22), were included in the analyses to avoid potential biases.

Enrollment occurred at the time of FGR diagnosis by means of obstetric ultrasound.

Data regarding maternal demographic characteristics, height and pregestational weight, pregestational body mass index (BMI, obtained from the ratio between the weight expressed in kilograms and the square of the height expressed in meters), pregestational body surface area (BSA, obtained by Boyd’s formula), obstetric history, mode of conception, cigarette smoking during pregnancy, prophylactic use of low-dose aspirin during pregnancy, and use of antihypertensive therapy were collected and registered in a dedicated log book which was periodically audited. Ultrasound data of the current pregnancy were also included in the log book. At recruitment, ultrasound fetal biometry and Doppler velocimetry of the uterine artery (UtA), umbilical artery (UA), and middle cerebral artery (MCA) were performed by an experienced physician, using standardized techniques. CPR was calculated as the ratio of MCA pulsatility index (PI) to UA-PI. The mean of the left and right UtA-PI was calculated and subsequently converted into multiples of the median (MoM) to adjust for gestational age in weeks (23).

At recruitment, patients also underwent a single hemodynamic investigation. Maternal brachial blood pressure (BP) was obtained using an upper arm automatic BP monitor, with the woman in a semi-recumbent position and using an appropriately sized cuff, and the mean arterial pressure (MAP) was calculated as (2 x diastolic BP + systolic BP)/3. Maternal hemodynamic indices were assessed using the USCOM-1A^®^ non-invasive device (ultrasound cardiac output monitor, USCOM Ltd, NSW, Australia), which has already been validated against echocardiography for use in pregnancy (24). USCOM-1A^®^ utilizes continuous-wave Doppler, with a non-imaging probe in the suprasternal notch, to obtain velocity-time integrals of transaortic blood flow at the left ventricular outflow tract. Using an internal anthropometric algorithm, which correlates the outflow tract diameter with the patient’s height, USCOM-1A^®^ multiplies the velocity-time integral by the aortic root diameter to calculate the stroke volume (SV). By measuring the time interval between each Doppler profile (cardiac cycle), heart rate (HR) can be calculated. Cardiac output (CO) and systemic vascular resistance (SVR) can also be calculated as follows: CO = SV x HR; SVR = MAP/CO. Participants remained in a semi-recumbent position and a small amount of conducting gel was applied to their skin at the level of the suprasternal notch. The Doppler probe was applied and moved through three dimensions to ensure that the velocity of blood was being measured at the left ventricular outflow tract and not in the more distal aorta. Each Doppler acquisition used for analysis had a minimum of two consecutive Doppler profiles (cardiac cycles). CO, SV, and SVR were converted into MoM to adjust for maternal phenotypic characteristics, active smoking in pregnancy, and gestational age in weeks at the time of assessment (25). Additional recorded hemodynamic measurements were Vpk, which is the peak velocity of the ventricular ejection, and the SMII, the Smith-Madigan Inotropy Index, which is a body surface area-indexed measure of the cardiac power. All hemodynamic evaluations were performed by adequately trained physicians, under standardized conditions, for the entire study cohort.

After recruitment, all women were followed until birth and data regarding pregnancy complications, gestational age at delivery, mode of birth, and perinatal outcomes were collected.

### Statistical Analyses

Data distribution was assessed using the Shapiro-Wilk test as well as graphical methods. Continuous variables were expressed through median and interquartile range, whereas categorical variables as relative and absolute frequencies. Statistical analyses were performed using the Chi Square test and Mann-Whitney U-Test.

We initially compared FGR cases according to the gestational age at diagnosis, distinguishing early- and late-onset FGR with the 32 gestational weeks cut-off. Sensitivity analyses including only cases of FGR with HDP (HDP-FGR) or isolated FGR (i-FGR) were also performed. Subsequently, cases of HDP-FGR were compared to cases of i-FGR, independent of gestational age at diagnosis.

In the second part of the analyses, we employed a Random Forest model with an automated feature selection by means of the Boruta algorithm to obtain a classificatory analysis to identify relevant variables in differentiating FGR cases (HDP-FGR versus i-FGR).

Specifically, we split the dataset into two parts, the training (70%) and the test (30%) set, to avoid overfitting. The Random Forest classification algorithm is an ensemble method based on decisions trees. Similar to bagging, Random Forest builds each tree on a bootstrap sample, but in addition to that, it considers a random subset of features for each tree split. The number of features randomly selected for the splits is the square root of 𝑝 where 𝑝 indicates the overall features number. In this way, the use of different bootstrap samples for different trees allows to reduce the risk of overfitting. In addition, the random features selection guarantees the decorrelation within trees. In turn, building a single tree to predict classes could cause overfitting, especially if the tree isn’t well pruned. The number of trees used for each Random Forest comparison is 500 and the number of features tried at each split depends on the number of important features obtained by the Boruta algorithm, which was 2 in the comparison HDP-FGR versus i-FGR.

Boruta is a feature selection algorithm. Precisely, it works as a wrapper algorithm around the Random Forest. Firstly, it adds randomness to the given data set by creating shuffled copies of all features (which are called shadow features). Then, it trains a Random Forest classifier on the extended data set and applies a feature importance measure (the default is Mean Decrease Accuracy) to evaluate the importance of each feature where a higher value means more important. At every iteration, it checks whether a real feature has a higher importance than the best of its shadow features (i.e., whether the feature has a higher Z score than the maximum Z score of its shadow features) and constantly removes features which are deemed highly unimportant. Finally, the algorithm stops either when all features get confirmed or rejected, or it reaches a specified limit of the Random Forest runs.

The α significance level was set at 5% (p-value < 0.05). Statistical analyses were performed using R software version 4, SPSS software version 28, and Prism software version 7.

## Results

During the study period, 146 pregnant women fulfilling the inclusion criteria were identified. Assessment of neonatal biometric data after birth led to exclusion of 44 cases with no evidence of neonatal growth restriction, thus leading to 102 patients in the final study population.

Cases were equally recruited at the three research sites: 31 (30.4%) at Monza, 33 (32.4%) at Milan, and 38 (37.2%) at Florence.

In 49 (48.1%) cases, FGR was associated to HDP, whereas 53 (51.9%) women were diagnosed with i-FGR. Also, 59 (57.8%) cases were diagnosed before 32 weeks’ gestation and were therefore identified as early-onset, with the remaining 43 (42.2%) cases recognized as late-onset FGR. In particular, 23/53 (43.4%) i-FGR cases versus 36/49 (73.5%) HDP-FGR cases were early-onset.

**Table 1** displays the results of the descriptive analysis regarding general characteristics, obstetric data, and perinatal outcomes of the entire study population.

**Table 1:** Demographic and clinical data of the entire study cohort.

Study population (*n* = 102)	
Maternal demographic characteristics	
Maternal age (years)	34.0 (29.0-38.0)
Caucasian ethnicity	84 (82.4)
Body mass index (kg/m^2^)	22.1 (19.9-24.9)
Diabetes mellitus	4 (3.9)
Pregestational hypothyroidism	7 (6.9)
Nulliparous	58 (56.9)
Spontaneous conception	93 (91.2)
Smoking in pregnancy	10 (9.8)
LDA in pregnancy	44 (43.1)
Antihypertensive drugs in pregnancy	34 (33.7)
GA at diagnosis of FGR (weeks)	31.2 (28.1-33.5)
**Pregnancy and fetal-neonatal outcomes**	
GDM	14 (13.7)
Gestational Hypertension	13 (12.7)
Preeclampsia	36 (35.3)
IUFD	1 (0.9)
GA at birth (weeks)	35.7 (32.3-37.4)
GA at birth <34 weeks	36 (35.3)
Cesarean section	74 (72.5)
Male gender	53 (52.0)
Birthweight (grams)	1770.0 (1295.0-2238.0)
Birthweight centile	4.0 (2.0-7.0)
Birthweight centile <3^rd^	32 (31.4)
NICU admission	64 (63.4)
Neonatal death	3 (3.0)

Data given as median (interquartile range) or number (%).

LDA, low dose aspirin; GA, gestational age; FGR, fetal growth restriction; GDM, gestational diabetes mellitus; IUFD, intrauterine fetal demise; UA, umbilical artery; NICU, Neonatal intensive care unit.

Smoking in pregnancy was defined as any active tobacco intake. Birthweight centile defined according to Italian neonatal charts, Ines charts (26).

The median gestational age (GA) at diagnosis of FGR was 31 weeks, and 35% of women gave birth before 34 weeks’ gestation, mostly by cesarean section. In addition, almost 65% of the newborns were admitted to the neonatal intensive care unit (NICU), and death occurred in three neonates.

Comparison between early- and late-onset FGR, including both normotensive pregnancies and those complicated by HDP, is shown in **Table 2**.

**Table 2:** Demographic, obstetric, and maternal hemodynamic data of early- versus late-onset FGR.

	FGR < 32 weeks *n *= 59	FGR ≥ 32 weeks *n* = 43	*p*-value
**Maternal characteristics and obstetric variables**			
Maternal age (years)	34 (29-38)	34 (29-37)	0.603
Nulliparous	30 (50.8)	28 (65.1)	0.163
Low dose ASA during pregnancy	26 (44.1)	18 (41.9)	0.842
Antihypertensive drugs in pregnancy	25 (42.4)	9 (20.9)	0.033
Active smoking in pregnancy	2 (3.4)	9 (20.9)	0.006
HDP	36 (61.0)	13 (30.2)	0.025
GA at birth (weeks)	32.4 (30.4-35.8)	37.3 (36.3-38.4)	0.180
GA at birth <34 weeks	34 (57.6)	2 (4.7)	<0.001
Birthweight centile <3^rd^	20 (33.9)	12 (27.9)	0.666
NICU admission	48 (82.8)*	16 (37.2)	<0.001
**Doppler velocimetry indices and hemodynamic assessment**			
GA at assessment (weeks)	30.4 (27.7-32.9)	36.0 (34.1-37.4)	<0.001
Mean UtA-PI MoM	1.67 (1.22-2.10)	1.19 (1.07-1.22)	0.002
Mean UtA-PI > 95^th^ centile	39 (66.1)	14 (32.5)	0.013
CPR < 5^th^ centile	27/49 (55.1)	13/42 (31.0)	0.034
Body mass index (kg/m^2^)	22.7 (19.9-24.9)	21.2 (19.5-24.9)	0.396
Body surface area (m^2^)	1.76 (1.71-1.87)	1.80 (1.69-1.87)	0.411
MAP (mmHg)	96.0 (90.0-105.0)	90.0 (81.0-97.0)	0.004
HR (bpm)	72.0 (63.0-85.0)	75.0 (63.0-90.0)	0.655
SV (mL)	65.0 (54.0-77.0)	69.0 (57.0-76.0)	0.773
SV MoM	0.82 (0.70-1.02)	0.86 (0.75-0.98)	0.253
SVR (dynes x s/cm^5^)	1567 (1356-1901)	1549 (1233-1926)	0.299
SVR MoM	1.49 (1.34-1.89)	1.48 (1.18-1.83)	0.198
CO (L/min)	4.8 (4.0-5.7)	4.8 (4.0-5.7)	0.985
CO MoM	0.78 (0.60-0.87)	0.76 (0.62-0.87)	0.459
Vpk (m/s)	1.2 (0.9-1.6)	1.3 (0.9-1.4)	0.610
SMII (W/m^2^)	1.5 (1.2-1.7)	1.4 (1.2-1.6)	0.133

Data given as median (interquartile range) or number (%).

FGR, fetal growth restriction; HDP, hypertensive disorders of pregnancy; ASA, aspirin; GA, gestational age; NICU, Neonatal intensive care unit; UtA-PI, uterine artery pulsatility index; CPR, cerebro-placental ratio; MAP, mean arterial blood pressure; HR, heart rate; SV, stroke volume; MoM, multiples of the median; SVR, systemic vascular resistance; CO, cardiac output; Vpk, peak velocity of flow profile; SMII, Smith-Madigan Inotropy Index.

Treatment with antihypertensive drugs was started after enrollment and non-invasive hemodynamic assessment were performed.

Birthweight centile defined according to Italian neonatal charts, Ines charts. (26).

*n=1 intrauterine fetal demise.

Women with early-onset growth restricted fetuses were more commonly diagnosed with HDP and treated with anti-hypertensive drugs; also, their newborns were more likely to be admitted to the NICU. In turn, pregnancies complicated by late-onset FGR had higher rates of active smoking during gestation.

Study recruitment coincided with FGR diagnosis, thus GA at assessment of early-onset FGR cases was lower than that of late-onset cases. Early-onset FGR cases also showed increased rates of abnormal maternal and fetal Doppler velocimetry parameters. Regarding the hemodynamic variables, only MAP differed between the two groups, with higher values in the early-onset FGR population, in line with the observed heightened prevalence of HDP.

Since HDP was more frequently identified among pregnant women with early-onset FGR and its presence is known to influence the maternal hemodynamic status (3), we performed sensitivity analyses of early- versus late-onset FGR evaluating HDP-FGR (**Table 3**) and i-FGR (**Table 4**) cases separately.

**Table 3:** Hemodynamic assessment among pregnancies complicated by HDP-FGR.

	HDP-FGR < 32 weeks *n *= 36	HDP-FGR ≥ 32 weeks *n *= 13	*p*-value
GA at assessment (weeks)	30.5 (27.8-33.1)	36.1 (32.9-37.0)	<0.001
Body mass index (kg/m^2^)	24.4 (21.9-26.6)	22.3 (19.6-29.4)	0.550
Body surface area (m^2^)	1.83 (1.71-1.96)	1.83 (1.79-2.10)	0.214
MAP (mmHg)	100.0 (93.0-107.0)	98.0 (95.0-108.0)	0.784
HR (bpm)	74.0 (64.0-86.5)	77.0 (63.5-102.0)	0.477
SV (mL)	63.0 (52.5-76.0)	60.0 (41.5-75.0)	0.391
SV MoM	0.81 (0.63-0.96)	0.73 (0.52-0.88)	0.152
SVR (dynes x s/cm^5^)	1810 (1467-2036)	1804 (1581-2221)	0.565
SVR MoM	1.62 (1.38-2.07)	1.85 (1.45-2.33)	0.157
CO (L/min)	4.6 (4.0-5.6)	4.3 (3.7-5.3)	0.456
CO MoM	0.72 (0.55-0.84)	0.62 (0.53-0.80)	0.195
Vpk (m/s)	1.1 (0.9-1.6)	0.9 (0.8-1.3)	0.215
SMII (W/m^2^)	1.4 (1.2-1.7)	1.2 (0.9-1.6)	0.220

Data given as median (interquartile range).

HDP-FGR, FGR associated with hypertensive disorders of pregnancy; GA, gestational age; MAP, mean arterial blood pressure; HR, heart rate; SV< stroke volume; MoM, multiples of the median; SVR, systemic vascular resistance; CO, cardiac output; Vpk, peak velocity of flow profile; SMII, Smith-Madigan Inotropy Index.

**Table 4:** Hemodynamic assessment in pregnant women complicated by i-FGR.

	iFGR < 32 weeks *n *= 23	iFGR ≥ 32 weeks *n* = 30	*p*-value
GA at assessment (weeks)	29.9 (27.0-32.3)	35.7 (34.3-37.6)	<0.001
Body mass index (kg/m^2^)	20.6 (18.0-22.8)	20.9 (19.5-23.5)	0.270
Body surface area (m^2^)	1.74 (1.58-1.77)	1.76 (1.68-1.83)	0.063
MAP (mmHg)	90.0 (80.0-97.0)	87.0 (79.5-93.0)	0.183
HR (bpm)	71.0 (62.0-85.0)	62.8 (52.0-73.5)	0.679
SV (mL)	69.0 (58.0-87.0)	70.5 (63.8-76.0)	0.911
SV MoM	0.85 (0.71-1.05)	0.90 (0.82-1.00)	0.288
SVR (dynes x s/cm^5^)	1395 (1201-1601)	1452 (1164-1702)	0.908
SVR MoM	1.38 (1.18-1.54)	1.33 (1.11-1.66)	0.313
CO (L/min)	5.5 (4.3-5.8)	4.9 (4.3-6.0)	0.820
CO MoM	0.81 (0.66-0.90)	0.79 (0.67-0.92)	0.445
Vpk (m/s)	1.3 (1.1-1.6)	1.4 (1.1-1.5)	0.534
SMII (W/m^2^)	1.5 (1.4-1.9)	1.4 (1.2-1.6)	0.130

Data shown as median (interquartile range).

i-FGR, isolated fetal growth restriction; GA, gestational age; MAP, mean arterial blood pressure; HR, heart rate; SV, stroke volume; MoM, multiples of the median; SVR, systemic vascular resistance; CO, cardiac output; Vpk, peak velocity of flow profile; SMII, Smith-Madigan Inotropy Index.

Similar to the analysis performed on the entire study cohort, early-onset FGR showed lower GA at diagnosis, whereas no differences regarding the hemodynamic parameters were identified between the two study groups among either HDP-FGR (**Table 3**) or i-FGR (**Table 4**).

We then compared the maternal hemodynamic status between pregnancies complicated by HDP-FGR and those complicated by i-FGR, independently of the GA at diagnosis with the 32-weeks cut-off. Results are shown in **Table 5**.

**Table 5:** Demographic, obstetric, and hemodynamic data of HDP-FGR and i-FGR.

	HDP-FGR *n *= 49	i-FGR *n* = 53	*p*-value
**Maternal characteristics and obstetric variables**			
Maternal age (years)	34 (29-38)	34 (29-37)	0.603
Active smoking during pregnancy	2 (4.1)	9 (16.9)	0.010
GA at birth (weeks)	32.4 (30.4-35.8)	37.3 (36.3-38.4)	0.180
GA at birth <34 weeks	23 (46.9)	13 (24.5)	0.023
Birthweight centile <3^rd^	12 (24.5)	20 (37.7)	0.200
NICU admission	40 (81.6)	24 (46.2)	<0.001
**Doppler velocimetry indices and hemodynamic assessment**			
GA at assessment (weeks)	32.0 (29.0-33.9)	33.4 (30.6-36.4)	0.058
Mean UtA-PI >95^th^ centile	33 (70.2)	19 (37.3)	0.001
CPR <5^th^ centile	25/44 (56.8)	15/47 (31.9)	0.021
Body mass index (kg/m^2^)	23.6 (20.9-26.6)	20.7 (18.4-23.1)	<0.001
Body surface area (m^2^)	1.83 (1.72-1.96)	1.74 (1.64-1.80)	<0.001
MAP (mmHg)	100.0 (93.0-107.0)	89.0 (80.0-93.5)	<0.001
HR (bpm)	76.0 (65.0-89.0)	73.0 (62.5-85.0)	1.000
SV (mL)	61.0 (52.0-76.0)	70.0 (60.0-80.0)	0.104
SV MoM	0.79 (0.62-0.90)	0.87 (0.79-1.04)	0.002
SVR (dynes x s/cm^5^)	1808 (1493-2048)	1395 (1194-1665)	<0.001
SVR MoM	1.68 (1.41-2.06)	1.38 (1.12-1.65)	<0.001
CO (L/min)	4.5 (3.9-5.5)	5.1 (4.3-5.8)	0.352
CO MoM	0.69 (0.55-0.84)	0.79 (0.66-0.90)	0.009
Vpk (m/s)	1.1 (0.8-1.5)	1.3 (1.1-1.5)	0.044
SMII (W/m^2^)	1.4 (1.1-1.7)	1.5 (1.2-1.7)	1.000

Data given as n (%) or median (interquartile range).

HDP-FGR, FGR associated with hypertensive disorders of pregnancy; i-FGR, isolated FGR; GA, gestational age; NICU, neonatal intensive care unit; UtA-PI, uterine artery pulsatility index; CPR, cerebro-placental ratio; MAP, mean arterial blood pressure; HR, heart rate; SV, stroke volume; MoM, multiples of the median; SVR, systemic vascular resistance; CO, cardiac output; Vpk, peak velocity of flow profile; SMII, Smith-Madigan Inotropy Index.

Birthweight centile defined according to Italian neonatal charts, ines charts (26).

HDP-FGR women displayed higher BMI and, accordingly, BSA values, than women with i-FGR. Interestingly, differences regarding the hemodynamic variables were identified, with HDP-FGR women showing increased SVR MoM and lower SV MoM, CO MoM, and Vpk values.

**Figure 1:**
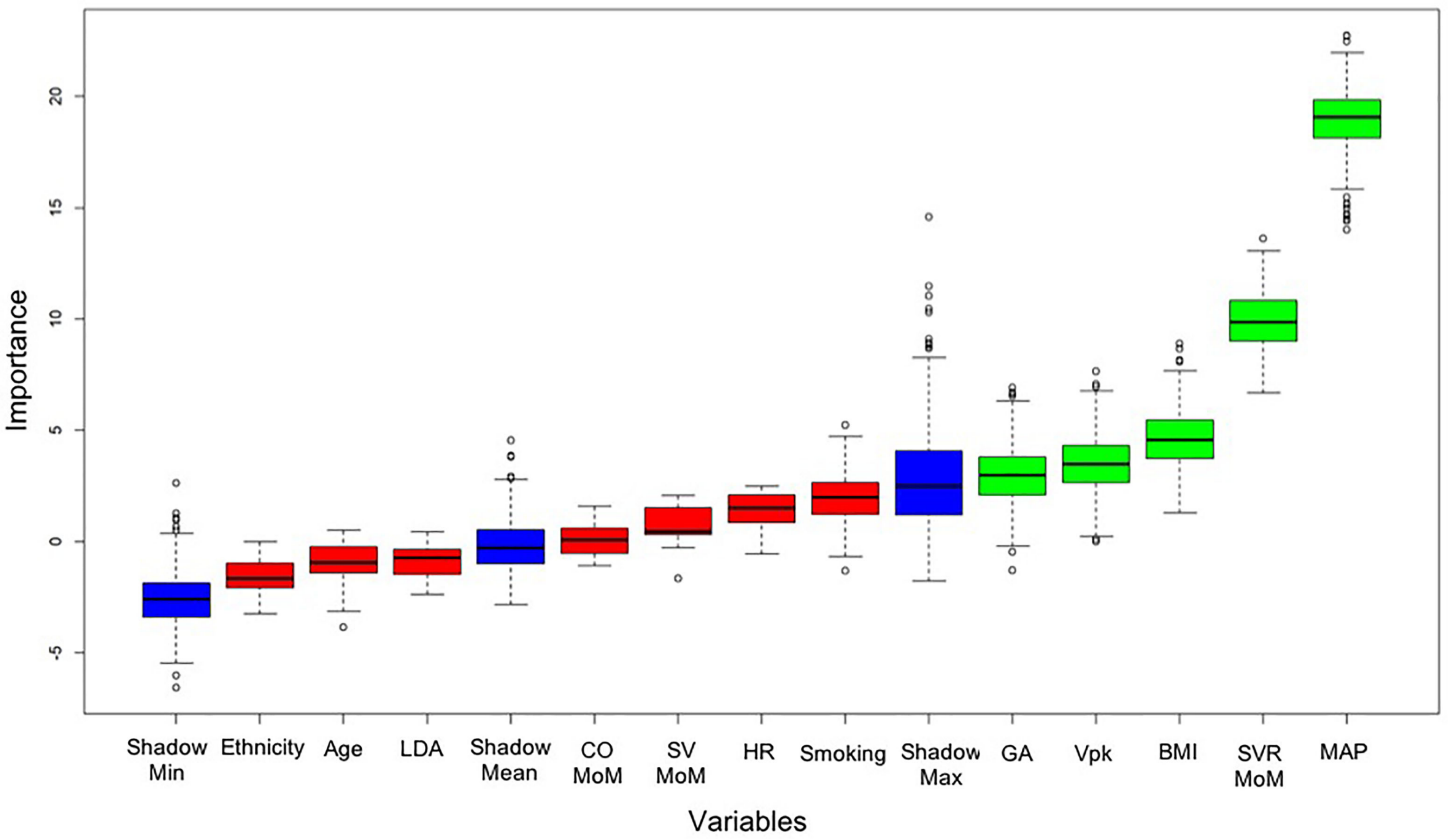
Classificatory analysis of FGR phenotypes. Important variables identified by the Boruta algorithm in the comparison between HDP-FGR and i-FGR cases. In decreasing order of importance, the Boruta algorithm identified MAP, SVR MoM, BMI, Vpk, and GA at diagnosis of FGR as the most important features with a discriminatory ability between the two FGR phenotypes.

**Figure 1** shows the classificatory analysis based on the Random Forest algorithm with an automated feature selection by means of the Boruta algorithm.

The classificatory ability of the Random Forest model showed an accuracy of 74.2%, a sensitivity of 87.5%, and a specificity of 60%, with a substantially significant overall performance (p=0.009). Five variables were identified significant for discriminating HDP-FGR from i-FGR cases, including MAP, SVR MoM, BMI, Vpk, and GA at FGR diagnosis.

## Discussion

Here we show that HDP, rather than GA at diagnosis of FGR, identifies specific maternal hemodynamic patterns that distinguish two different FGR phenotypes. In addition, our data show that maternal hemodynamics, alongside maternal phenotypic characteristics, such as BMI, play a central role in classifying these high-risk pregnancies and differentiating HDP-FGR from i-FGR cases.

The recently published ISUOG practice guidelines on FGR have incorporated the diagnostic criteria derived from an international Delphi survey consensus, which recognizes two main FGR phenotypes according to the timing of diagnosis: early-onset if before 32 weeks of gestation and late-onset if at or after this temporal cut-off (3, 4). These phenotypes differ in natural history, Doppler findings, adverse perinatal outcomes, and management. Of note, the ISUOG guidelines have for the first time included the maternal hemodynamic status in this classification, suggesting a more marked hemodynamic impairment characterized by low CO and high SVR in early- versus late-onset FGR (3).

Several studies have investigated maternal hemodynamics among pregnancies complicated by FGR and small for gestational age (SGA) fetuses, identifying substantial differences between these two conditions (27–29). In turn, only one research work has been published so far focusing on the hemodynamic status of FGR as defined by the international Delphi survey consensus and comparing early- to late-onset cases (28). The authors did not recognize hemodynamic differences between early- and late-onset FGR, although the study was limited by the small size of each study group.

In line with these findings and supported by the assessment of a larger group of pregnancies complicated by FGR, we did not identify any difference in the hemodynamic status between early- and late-onset FGR, except for higher MAP values in early-onset cases, which were more frequently associated with HDP (3, 4). Similar results were obtained when performing sensitivity analyses including only HDP-FGR or i-FGR cases. In contrast, substantial differences in maternal hemodynamic parameters were recognized when HDP-FGR were compared to i-FGR, independently of the GA at diagnosis. In particular, hypertensive women with a growth restricted fetus showed a substantially increased hemodynamic impairment compared to normotensive FGR pregnant patients, with higher values of MAP and SVR MoM and lower values of SV MoM, CO MoM, and Vpk. These data are in line with previous publications, although using a different technique for hemodynamic evaluation and inadequate definition of FGR (13, 14, 30).

Altogether these findings suggest that the presence or absence of HDP with an underlying diagnosis of FGR, rather than the simple temporal cut-off of 32 gestational weeks at FGR diagnosis, is a more accurate criterion to highlight differences from the point of view of maternal hemodynamics, thus distinguishing FGR phenotypes likely requiring different therapeutic management (31).

We also addressed the classificatory issue of FGR phenotypes by means of a Random Forest algorithm with an automated feature selection using the Boruta algorithm. These analyses allowed us to evaluate significant variables with the potential for discriminating HDP-FGR from i-FGR cases. Of note, five features were identified, which were, in order of importance, MAP, SVR MoM, BMI, Vpk, and GA at FGR diagnosis. While the inclusion of MAP seems obvious, the identification of the other above-mentioned features is less straightforward and of major importance, since it highlights the relevance of maternal hemodynamic status and phenotype, alongside the timing at FGR diagnosis, in discriminating different FGR phenotypes. The system displayed an overall good classificatory ability (p=0.009) with a 74.2% accuracy, an 87.5% sensitivity, and a 60.0% specificity.

The strengths of our study are several. First, the study design, a multicenter prospective study conducted at three maternal-fetal referral centers. Second, the large size of the study groups, which allowed us to perform robust analyses on the entire cohort as well as on the study subgroups, such as HDP-FGR and i-FGR. Third, the use of a strict definition of FGR according to the recent international Delphi consensus diagnostic criteria (4), thus limiting potential biases related to inclusion of SGA fetuses. Finally, the evaluation of maternal hemodynamic status by a small group of trained physicians at each research site.

Our study is not without limitations. In particular, USCOM-1A evaluation was performed only once at the time of FGR diagnosis, thus preventing us to identify potential hemodynamic changes antecedent to FGR onset or subsequent to FGR diagnosis and possibly reflecting worsening of fetal status requiring delivery.

## Conclusions

Our work supports the hypothesis that the presence of HDP, rather than the mere temporal variable of GA at FGR diagnosis, is a more accurate criterion to appreciate specific maternal hemodynamic patterns associated with FGR and to adequately distinguish different FGR phenotypes. Of note, such findings are in line with previously published data on HDP (11, 17, 18) and could be relevant in guiding a tailored approach for management of these high-risk pregnancies, leading towards a more effective and accurate precision medicine in this field of obstetrics (31–35).

In addition, the classificatory analysis based on the Random Forest algorithm has shown that the classification of these high-risk pregnancies should include not only a single parameter, such as the GA at FGR diagnosis, but several variables, among which those related to maternal phenotype, i.e., BMI, and hemodynamic status are the most relevant.

## Data Availability Statement

The raw data supporting the conclusions of this article will be made available by the authors, without undue reservation.

## Ethics Statement

The studies involving human participants were reviewed and approved by University of Milan-Bicocca, IRB n. 2988, approved on December 13, 2018. The patients/participants provided their written informed consent to participate in this study.

## Author Contributions

All the above listed authors have contributed to the study design, data collection, data analyses, writing of the first version of the manuscript, and revision of the final version of the manuscript.

## Conflict of Interest

The authors declare that the research was conducted in the absence of any commercial or financial relationships that could be construed as a potential conflict of interest.

## Publisher’s Note

All claims expressed in this article are solely those of the authors and do not necessarily represent those of their affiliated organizations, or those of the publisher, the editors and the reviewers. Any product that may be evaluated in this article, or claim that may be made by its manufacturer, is not guaranteed or endorsed by the publisher.

## References

[1] Unterscheider J Daly S Geary MP Kennelly MM McAuliffe FM O'Donoghue K. Optimizing the Definition of Intrauterine Growth Restriction: The Multicenter Prospective PORTO Study. Am J Obstetrics Gynecol (2013) 208:290.e1–6. doi: 10.1097/OGX.0b013e3182a0597f 23531326

[2] Lees CC Marlow N van Wassenaer-Leemhuis A Arabin B Bilardo CM Brezinka C. 2 Year Neurodevelopmental and Intermediate Perinatal Outcomes in Infants With Very Preterm Fetal Growth Restriction (TRUFFLE): A Randomised Trial. (2015) 385:2162–72. doi: 10.1016/S0140-6736(14)62049-3 25747582

[3] Lees CC Stampalija T Baschat A da Silva Costa F Ferrazzi E Figueras F. ISUOG Practice Guidelines: Diagnosis and Management of Small-for-Gestational-Age Fetus and Fetal Growth Restriction. Ultrasound Obstet Gynecol (2020) 56:298–312. doi: 10.1002/uog.22134 32738107

[4] Gordijn SJ Beune IM Thilaganathan B Papageorghiou A Baschat AA Baker PN. Consensus Definition of Fetal Growth Restriction: A Delphi Procedure. Ultrasound Obstet Gynecol (2016) 48:333–9. doi: 10.1002/uog.15884 26909664

[5] Mifsud W Sebire NJ. Placental Pathology in Early-Onset and Late-Onset Fetal Growth Restriction. Fetal Diagnosis Ther (2014) 36:117–28. doi: 10.1159/000359969 24577279

[6] Duvekot JJ Cheriex EC Pieters FA Menheere PP Schouten HJ Peeters LL. Maternal Volume Homeostasis in Early Pregnancy in Relation to Fetal Growth Restriction. Obstetrics Gynecol (1995) 85:361–7. doi: 10.1016/0029-7844(94)00417-C 7862373

[7] Foo FL Mahendru AA Masini G Fraser A Cacciatore S MacIntyre DA. Association Between Prepregnancy Cardiovascular Function and Subsequent Preeclampsia or Fetal Growth Restriction. Hypertension (2018) 72:442–50. doi: 10.1161/HYPERTENSIONAHA.118.11092 29967040

[8] Ghossein-Doha C Hooijschuur MCE Spaanderman MEA. Pre-Eclampsia: A Twilight Zone Between Health and Cardiovascular Disease? J Am Col Cardio (2018) 72:12–6. doi: 10.1016/j.jacc.2018.04.049 29957220

[9] Rang S van Montfrans GA Wolf H. Serial Hemodynamic Measurement in Normal Pregnancy, Preeclampsia, and Intrauterine Growth Restriction. Am J Obstetrics Gynecol (2008) 198:519.e1–9. doi: 10.1016/j.ajog.2007.11.014 18279824

[10] Salas SP Rosso P Espinoza R Robert JA Valdés G Donoso E. Maternal Plasma Volume Expansion and Hormonal Changes in Women With Idiopathic Fetal Growth Retardation. Obstetrics Gynecol (1993) 81:1029–33. 8497346

[11] Tay J Masini G McEniery CM Giussani DA Shaw CJ Wilkinson IB. Uterine and Fetal Placental Doppler Indices are Associated With Maternal Cardiovascular Function. Am J Obstetrics Gynecol (2019) 220:96.e1–.e8. doi: 10.1016/j.ajog.2018.09.017 30243605

[12] Thilaganathan B. Placental Syndromes: Getting to the Heart of the Matter. Ultrasound Obstet Gynecol (2017) 49:7–9. doi: 10.1002/uog.17378 28067440

[13] Bamfo JE Kametas NA Chambers JB Nicolaides KH. Maternal Cardiac Function in Fetal Growth-Restricted and Non-Growth-Restricted Small-for-Gestational Age Pregnancies. Ultrasound Obstet Gynecol (2007) 29:51–7. doi: 10.1002/uog.3901 17200990

[14] Melchiorre K Sutherland GR Liberati M Thilaganathan B. Maternal Cardiovascular Impairment in Pregnancies Complicated by Severe Fetal Growth Restriction. Hypertension (2012) 60:437–43. doi: 10.1161/HYPERTENSIONAHA.112.194159 22733460

[15] Vasapollo B Valensise H Novelli GP Altomare F Galante A Arduini D. Abnormal Maternal Cardiac Function Precedes the Clinical Manifestation of Fetal Growth Restriction. Ultrasound Obstet Gynecol (2004) 24:23–9. doi: 10.1002/uog.1095 15229912

[16] Mecacci F Avagliano L Lisi F Clemenza S Serena C Vannuccini S. Fetal Growth Restriction: Does an Integrated Maternal Hemodynamic-Placental Model Fit Better? Reprod Sci (2021) 28:2422–35. doi: 10.1007/s43032-020-00393-2 PMC8346440 33211274

[17] Ferrazzi E Stampalija T Monasta L Di Martino D Vonck S Gyselaers W. Maternal Hemodynamics: A Method to Classify Hypertensive Disorders of Pregnancy. Am J Obstetrics Gynecol (2018) 218:124.e1–.e11. doi: 10.1016/j.ajog.2017.10.226 29102503

[18] Masini G Foo LF Tay J Wilkinson IB Valensise H Gyselaers W. Preeclampsia has Two Phenotypes Which Require Different Treatment Strategies. Am J Obstetrics Gynecol (2022) 226(2S):S1006–S1018. doi: 10.1016/j.ajog.2021.09.006 34774281

[19] Ferrazzi E Zullino S Stampalija T Vener C Cavoretto P Gervasi MT. Bedside Diagnosis of Two Major Clinical Phenotypes of Hypertensive Disorders of Pregnancy. Ultrasound Obstet Gynecol (2016) 48:224–31. doi: 10.1002/uog.15741 26350023

[20] Brown MA Magee LA Kenny LC Karumanchi SA McCarthy FP Saito S. Hypertensive Disorders of Pregnancy: ISSHP Classification, Diagnosis, and Management Recommendations for International Practice. Hypertension (2018) 72:24–43. doi: 10.1161/HYPERTENSIONAHA.117.10803 29899139

[21] Beune IM Bloomfield FH Ganzevoort W Embleton ND Rozance PJ van Wassenaer-Leemhuis AG. Consensus Based Definition of Growth Restriction in the Newborn. J Pediatr (2018) 196:71–6.e1. doi: 10.1016/j.jpeds.2017.12.059 29499988

[22] Zullino S Di Martino D Stampalija T Mecacci F Ferrazzi E. Prenatal Assessment of Fetal Growth Restriction (FGR) Versus Post-Natal Diagnosis of Small for Gestational Age (SGA) Based on Newborns Weight Charts. Arch Obstetrics Gynecol (2021) 2:121–2.

[23] Gómez O Figueras F Fernández S Bennasar M Martínez JM Puerto B. Reference Ranges for Uterine Artery Mean Pulsatility Index at 11-41 Weeks of Gestation. Ultrasound Obstet Gynecol (2008) 32:128–32. doi: 10.1002/uog.5315 18457355

[24] Vinayagam D Patey O Thilaganathan B Khalil A. Cardiac Output Assessment in Pregnancy: Comparison of Two Automated Monitors With Echocardiography. Ultrasound Obstet Gynecol (2017) 49:32–8. doi: 10.1002/uog.15915 26970353

[25] Vinayagam D Thilaganathan B Stirrup O Mantovani E Khalil A. Maternal Hemodynamics in Normal Pregnancy: Reference Ranges and Role of Maternal Characteristics. Ultrasound Obstet Gynecol (2018) 51:665–71. doi: 10.1002/uog.17504 28437601

[26] Bertino E Spada E Occhi L Coscia A Giuliani F Gagliardi L. Neonatal Anthropometric Charts: The Italian Neonatal Study Compared With Other European Studies. J Pediatr Gastroenterol Nutr (2010) 51:353–61. doi: 10.1097/MPG.0b013e3181da213e 20601901

[27] Di Pasquo E Ghi T Dall'Asta A Angeli L Ciavarella S Armano G. Hemodynamic Findings in Normotensive Women With Small-for-Gestational-Age and Growth-Restricted Fetuses. Acta Obstetricia Gynecol Scand (2021) 100:876–83. doi: 10.1111/aogs.14026 33084031

[28] Farsetti D Pometti F Tiralongo GM Lo Presti D Pisani I Gagliardi G. Distinction Between SGA and FGR by Means of Fetal Umbilical Vein Flow and Maternal Hemodynamics. J Matern Fetal Neonatal Med (2021), 1–7. doi: 10.1080/14767058.2021.1918091 33938366

[29] Perry H Lehmann H Mantovani E Thilaganathan B Khalil A. Are Maternal Hemodynamic Indices Markers of Fetal Growth Restriction in Pregnancies With a Small-for-Gestational-Age Fetus? Ultrasound obstetrics gynecol Off J Ultrasound Obstet Gynecol (2020) 55:210–6. doi: 10.1002/uog.20419 31381215

[30] Di Martino DD Ferrazzi E Garbin M Fusè F Izzo T Duvekot J. Multivariable Evaluation of Maternal Hemodynamic Profile in Pregnancy Complicated by Fetal Growth Restriction: Prospective Study. Ultrasound Obstet Gynecol (2019) 54:732–9. doi: 10.1002/uog.20118 30207002

[31] Vasapollo B Novelli GP Valensise H. Hemodynamic Guided Treatment of Hypertensive Disorders in Pregnancy: Is It Time to Change Our Mind? J Matern Fetal Neonatal Med (2021) 34:3830–1. doi: 10.1080/14767058.2019.1695771 31771371

[32] McLaughlin K Scholten RR Kingdom JC Floras JS Parker JD. Should Maternal Hemodynamics Guide Antihypertensive Therapy in Preeclampsia? Hypertension (2018) 71:550–6. doi: 10.1161/HYPERTENSIONAHA.117.10606 29437898

[33] Stott D Papastefanou I Paraschiv D Clark K Kametas NA. Serial Hemodynamic Monitoring to Guide Treatment of Maternal Hypertension Leads to Reduction in Severe Hypertension. Ultrasound Obstet Gynecol (2017) 49:95–103. doi: 10.1002/uog.17341 27800645

[34] Valensise H Farsetti D Pisani I Tiralongo GM Lo Presti D Gagliardi G. Friendly Help for Clinical Use of Maternal Hemodynamics. J Matern Fetal Neonatal Med (2021) 34:3075–9. doi: 10.1080/14767058.2019.1678136 31619097

[35] Phillips RA Ma Z Kong B Gao L. Maternal Hypertension, Advanced Doppler Haemodynamics and Therapeutic Precision: Principles and Illustrative Cases. Curr Hypertension Rep (2020) 22:49. doi: 10.1007/s11906-020-01060-2 PMC7359153 32661569

